# Sublethal abamectin exposure alters intraguild predation and population structure of predatory mites

**DOI:** 10.1007/s10646-026-03159-9

**Published:** 2026-09-26

**Authors:** Bruna R. M. Campelo, Ana J. D. Monteiro, José W. da S. Melo, e. Debora B. Lima

**Affiliations:** Department of Zoology, Centro de Biociências, Universidade Federal de Pernambuco, Programa de Pós-graduação em Biologia Animal, Av. Professor Moraes Rego, 1235 - Cidade Universitária, 50670-901 Recife, PE Brasil

**Keywords:** Ecotoxicology, Interaction, Pesticide, Phytoseiidae

## Abstract

Chemical contaminants can disrupt predator-prey dynamics in agroecosystems, particularly through intraguild predation (IGP), a key interaction influencing population structure and dynamics. The objective of this study was to investigate intraguild predation between *Amblyseius largoensis* (Muma) and *Euseius alatus* DeLeon (Mesostigmata: Phytoseiidae), with particular emphasis on how an extraguild food resource (pollen), sublethal exposure to the acaricide abamectin, and their combined effects influence predator feeding behavior, oviposition, and population dynamics. Adult females of both predators were exposed to intraguild prey (eggs and larvae) in the presence or absence of extraguild food (pollen) and abamectin residues. In addition, population-level experiments were conducted to evaluate long-term interaction outcomes. Experimental results revealed that both feeding activity and oviposition of the two predator species were significantly affected by the presence of pesticide residues and the availability of pollen as an extraguild food source. Population dynamics assays indicated that *A. largoensis* was the dominant species; however, exposure to pesticide residues reduced the longevity of both predator populations.

## Introduction

Lethal effects of pesticides are frequently reported in natural enemies (Lima et al. 2016; Barros et al. 2022). However, in addition to their direct lethal effects, chemical contaminants can disrupt interspecific interactions, altering population stability and ecosystem functions (Guedes et al. 2016; Lima et al. 2015). Understanding these indirect pathways is fundamental to ecotoxicology because contaminants may affect ecological processes beyond direct mortality. By altering species interactions such as predation, competition, and intraguild predation, chemical stressors can influence population dynamics, community structure, and ecosystem functions, generating consequences that may not be detected through conventional toxicity assessments focused solely on individual survival.

Intraguild predation (IGP) is an ecological interaction that combines aspects of both predation and competition, occurring when one species consumes another that utilizes the same food resources (Polis and Holt, 1992). This type of interaction has attracted increasing attention due to its ecological and evolutionary implications (Polis et al. 1989; Holt and Polis, 1997; Schausberger and Croft, 2000; Janssen et al. 2007; Walzer and Schausberger, 2013; Marques et al. 2018; Momen and Abdel-Khalek, 2021). IGP is widely distributed across different taxonomic groups, can occur at multiple trophic levels, and exerts significant influence on community structure, species abundance and distribution, and the evolution of the interacting species (Polis et al. 1989; Mylius et al. 2001; Montserrat et al. 2012; Toscano et al. 2017; Farazmand and Fathipour, 2026).

Studies of IGP are particularly relevant in agricultural systems that rely on the services of biological control agents, especially among predators that share the same pest species. Predator coexistence may lead to direct competition or predation among predators, potentially compromising the effectiveness of biological control (Rosenheim et al. 1995; Rosenheim and Harmon, 2006). A notable example of coexistence has been documented between the predatory mites *Amblyseius largoensis* (Muma) and *Euseius alatus* DeLeon (Mesostigmata: Phytoseiidae), which naturally occur on coconut palm (*Cocos nucifera* L. (Arecales: Arecaceae) leaflets and may contribute to the regulation of *Aceria guerreronis* Keifer (Trombidiformes: Eriophyidae), a key pest of coconut palms (Galvão et al. 2007; Lawson-Balagbo et al. 2008; Melo et al. 2015). Although the coexistence of these species has been well documented (Lawson-Balagbo et al. 2008; Reis et al. 2008; Melo et al. 2015), aspects of IGP between them, as well as the ecological and extrinsic factors influencing their persistence or exclusion in natural environments, remain poorly understood, particularly under pesticide exposure.

Interactions among predators can be shaped by intrinsic factors of the organisms involved, such as life stage, dietary specialization, and body size (Claessen et al. 2002), as well as by extrinsic factors, such as toxic substances or contaminants and the presence of extraguild food (e.g. pollen) (Wright and Verkerk, 1995; Calabuig et al. 2018). Importantly, the trophic strategies of these predators differ: most *Euseius* species, including *E. alatus*, are type IV predators that feed preferentially on pollen, whereas *A. largoensis* is considered a type III predator, more dependent on animal prey (McMurtry et al. 2013). These differences may strongly affect IGP outcomes when pollen is available.

In this context, agricultural systems represent particularly suitable environments for investigating how environmental and anthropogenic factors modulate ecological interactions. In many cropping systems, biological control provided by natural enemies is an important component of pest management and is frequently combined with pesticide applications. This is especially relevant in coconut production, where the coconut mite, *A. guerreronis*, is one of the most economically important pests, causing substantial reductions in fruit quality and yield and resulting in significant economic losses (Rezende et al. 2016). Predatory mites such as *A. largoensis* and *E. alatus* have been identified as potential biological control agents of this pest (Galvão et al. 2007; Melo et al. 2015). However, their activity frequently overlaps with the application of abamectin, one of the most widely used acaricides for the management of *A. guerreronis* (Rezende et al. 2016). Abamectin belongs to the avermectin–milbemycin group (IRAC Group 6) and acts as a glutamate-gated chloride channel (GluCl) activator (Yu, 2008; IRAC, 2025). Although highly effective against eriophyid mites, sublethal exposure of non-target predatory mites may impair behavior, reproduction, and predator–predator interactions, potentially compromising biological control (Lima et al. 2013a, 2015; Barros et al. 2022). Such systems therefore provide an opportunity to examine how ecological interactions among predators are modified by both extraguild food availability and pesticide exposure.

In agricultural systems where biological control and pesticide applications coexist, the outcome of intraguild predation may be influenced by both resource availability and chemical stressors. Based on the distinct trophic strategies of *A. largoensis* and *E. alatus*, as well as the known effects of pesticides on predator behavior and reproduction, we hypothesized that (i) the availability of extraguild food (pollen) would reduce the intensity of intraguild predation, particularly for *E. alatus*, which relies heavily on pollen resources; (ii) sublethal exposure to abamectin would alter predation and oviposition rates, thereby affecting population dynamics; and (iii) these effects would influence the outcome of species coexistence. To test these hypotheses, we investigated intraguild predation between *A. largoensis* and *E. alatus*, focusing on the influence of extraguild food (pollen), sublethal exposure to abamectin, and the combined effects of these factors on predator consumption, oviposition, and population dynamics. Accordingly, the objective of this study was to investigate how extraguild food (pollen), sublethal exposure to abamectin, and their combined effects influence intraguild predation, oviposition, and population dynamics between *A. largoensis* and *E. alatus*.

## Materials and methods

### Collection and rearing of predatory mites

Colonies of the predatory mites *A. largoensis* and *E. alatus* were initiated with approximately 100 individuals of each species, collected from coconut palm (*Cocos nucifera*) leaflets or cashew (*Anacardium occidentale* L. (Sapindales: Anacardiaceae) plants. Separate rearing units were established for each species. For *A. largoensis*, the units consisted of black PVC discs (13 cm diameter, 1 mm thickness), whereas for *E. alatus*, cotyledonary leaves of jack bean (*Canavalia ensiformis* (L.) DC. (Fabales: Fabaceae), 13 cm in diameter with the abaxial surface facing upward, were used. Both the PVC discs and jack bean leaves were placed on top of a filter paper disc (15 cm diameter), which was set on a polyethylene foam pad (15 cm diameter, 1 cm thickness) kept moist with distilled water. The periphery of the PVC discs and jack bean leaves was lined with strips of moistened hydrophilic cotton to prevent mite escape and to provide an additional water source. A tuft of cotton covered with a small glass cover slip (1 cm²) was placed in the center of each unit to serve as a shelter and oviposition site for the mites. The predators were fed with coconut palm pollen (renewed every two days), 10% honey solution, and *Tetranychus urticae* Koch (Trombidiformes: Tetranychidae). The rearing units were maintained in a controlled laboratory environment at 27 ± 1 °C, 75% RH, and a 12:12 h (L:D) photoperiod.

### Pesticide

The pesticide used was abamectin (Abamectin 72 EC, 72 g a.i. L⁻¹, Nortox) at 3.375 mg a.i. L⁻¹ (25% of the recommended field dose). This concentration was defined based on preliminary acute mortality tests (24 h), which showed partial survival in both predator species, allowing the assessment of comparable sublethal effects. This concentration reflects environmentally relevant levels expected on coconut leaflets after field spraying. It is plausible that, under field conditions, these predators may be exposed to pesticide residues at levels similar to those tested here, either through contact with treated plant surfaces after spraying and/or during the degradation process of the pesticide. Thus, this concentration reflects environmentally relevant levels expected on coconut leaflets after field spraying.

### Effects of pesticide residues and extraguild food (pollen) on intraguild predation and oviposition of the predators

**Fig. 1:**
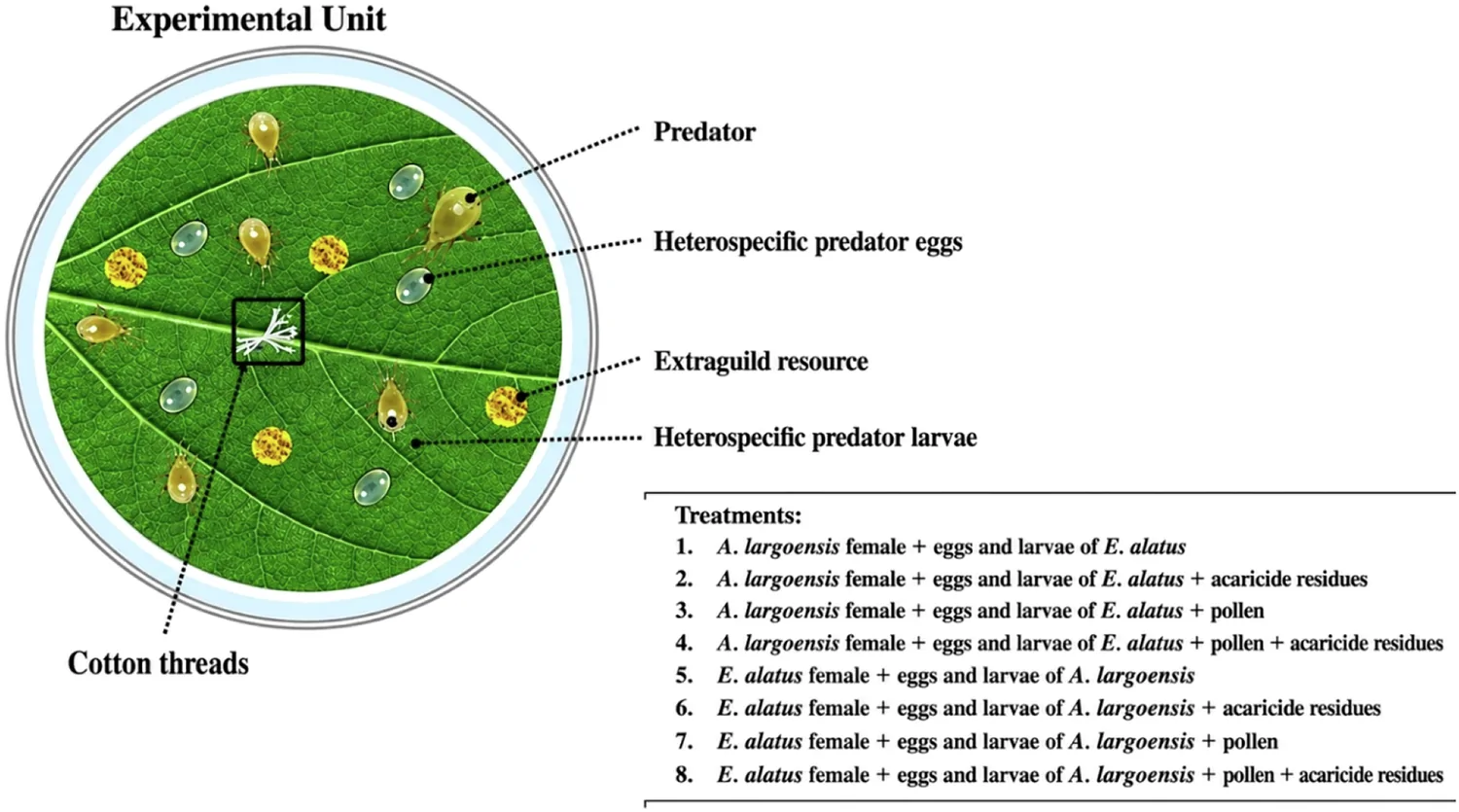
Experimental unit and treatments used in the study

The experimental units consisted of discs of cotyledonary leaves of jack bean (*C. ensiformis*), 8 cm in diameter, with the abaxial surface facing upward. Each leaf disc was placed on a filter paper disc of the same diameter, which rested on a polyethylene foam pad (8 cm diameter, 1 cm thickness) inside a Petri dish (9 cm diameter, 2 cm height). The periphery of the jack bean leaf was lined with strips of moistened hydrophilic cotton to prevent mite escape and to provide a source of moisture. A tuft of cotton covered with a small glass cover slip (0.5 cm²) was placed at the center of the experimental unit to serve as a shelter for the predators (Fig. 1).

Jack bean leaf discs were immersed for 5 s in 50 mL solutions of the pesticide or in distilled water (control), following Method 004 proposed by the Insecticide Resistance Action Committee (IRAC) (www.irac-online.org). After drying (30 min), each unit received: One adult predator female (pre-conditioned by 3 h of starvation, 2–3 days old post-emergence, and mated); Five eggs and five larvae of intraguild prey (collected from colonies maintained on untreated leaves and standardized on the same day); and Presence or absence of coconut pollen (0.0014 g). The number of intraguild prey (five eggs and five larvae) was selected based on previous studies of intraguild predation in phytoseiid mites (Walzer and Schausberger, 1999; Montserrat et al. 2006); and to provide sufficient prey availability throughout the 24-h experimental period. Pollen was used as the extraguild food source because it represents a naturally available resource in coconut palms, where *A. largoensis* and *E. alatus* coexist. Although *T. urticae* is commonly used as prey for laboratory rearing of phytoseiid mites, it is not associated with coconut palms under natural conditions (Lawson-Balagbo et al. 2008). Therefore, pollen was selected to better simulate the ecological context in which these predator species interact in the field.

The numbers of eggs and larvae consumed, as well as predator eggs laid, were recorded 24 h after the predators were introduced into the experimental units. Mortality was also recorded throughout the 24-h assay. Because the objective of this experiment was to evaluate the sublethal effects of abamectin on predation and oviposition, only females that survived until the end of the experimental period were included in the analyses. Experimental units in which predator mortality occurred were replaced to maintain the planned number of replicates. Cannibalism among intraguild prey in the absence of pollen was also monitored but was not observed during the experimental period.

The effects of pesticide residues and extraguild food (pollen) on intraguild predation and oviposition were evaluated separately for *A. largoensis* and *E. alatus*. Within each predator species, the experiment followed a 2 × 2 factorial design, with pesticide residues (presence or absence) and pollen (presence or absence) as fixed factors (Fig. 1).

Statistical analyses were performed in R (R Core Team, 2025) using the ARTool package. For each predator species and response variable, normality was assessed using the Shapiro–Wilk test. Because the data deviated significantly from normality, the effects of pesticide residues, pollen, and their interaction were analyzed using aligned rank transform (ART) ANOVA. When significant effects were detected, pairwise comparisons were performed using estimated marginal means (EMMs) with Tukey’s adjustment.

### Effects of pesticide residues on the population dynamics of predators in arenas

The experimental units were similar to those used for rearing *E. alatus*, consisting of jack bean cotyledonary leaves placed on a moist foam pad inside plastic trays. Unlike the rearing units, however, the leaves were previously treated with either abamectin or distilled water (control). Ten adult females of each predator species were released simultaneously into each experimental arena in the presence of coconut pollen (0.011 g), which was replenished every two days. The experiments were conducted until at least one predator species became extinct (or both species under pesticide treatment).

Populations were recorded every two days. Species identification was based on morphological traits (Z5 seta, body shape, coloration (Lofego et al. 2024), with confirmation by slide mounting at the end of the trials. The experimental units were maintained in the laboratory under controlled conditions (27 ± 1 °C, 75% RH, and a 12 h photoperiod). Each treatment was replicated 20 times.

Statistical analyses were performed in R software (version 5.4.1) using the *nlme* and *emmeans* packages. A linear mixed-effects model (LME) was fitted with the *lme* function, including treatment, time, and their interaction as fixed factors, and arena as a random factor to account for repeated measures. The response variable (number of individuals) was log-transformed [log(Num + 1)] to meet the assumptions of normality and homoscedasticity. The significance of the effects was assessed by ANOVA, and the Treatment × Time interaction was significant (p < 0.05), indicating variation in treatment effects over time. Estimated marginal means (EMMs) were computed with the *emmeans* package, and multiple comparisons among treatments were performed within each time point using Tukey-adjusted p-values.

## Results

### Effects of pesticide residues and extraguild food (pollen) on intraguild predation and oviposition of the predators

**Fig. 2:**
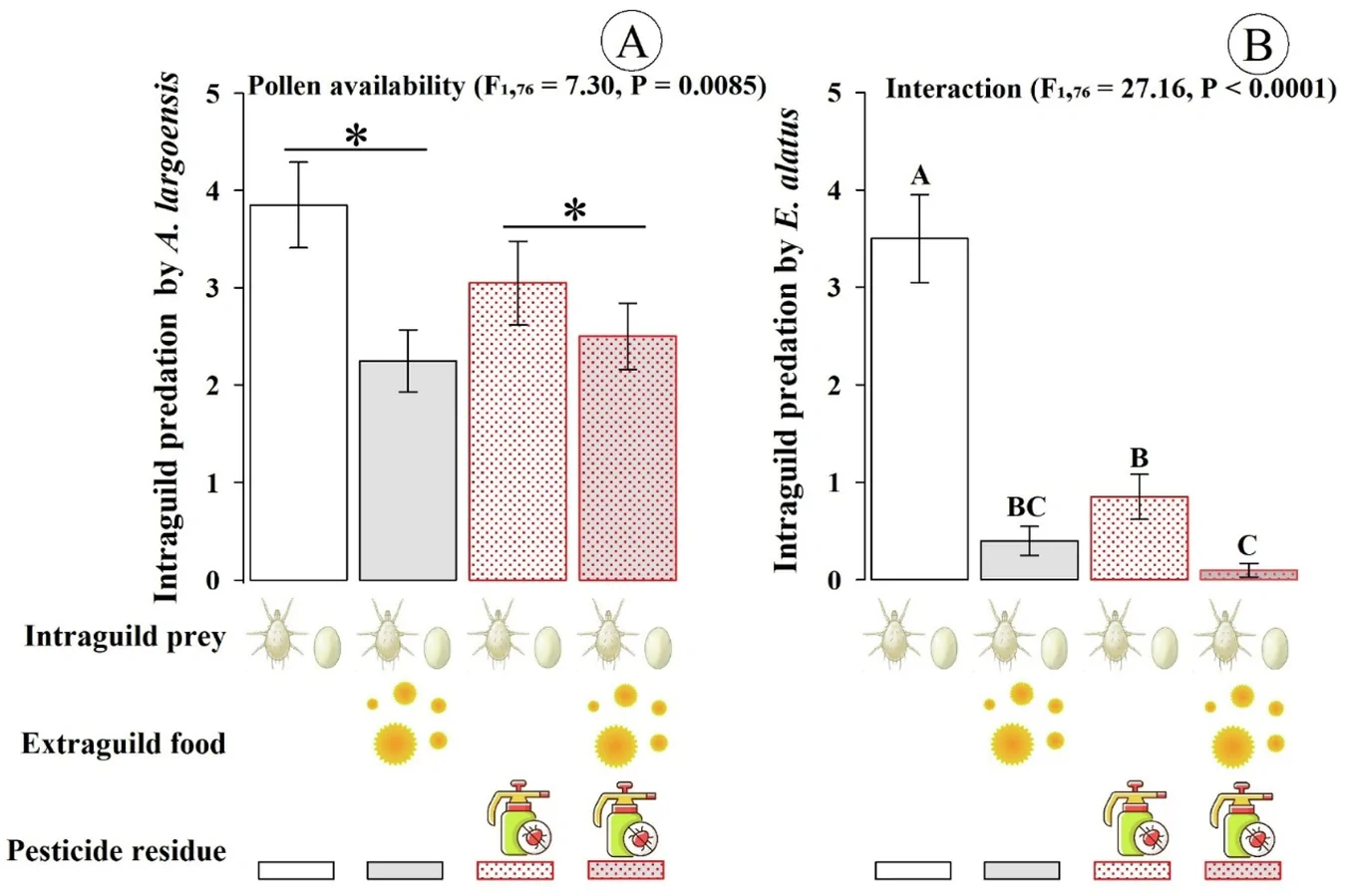
Intraguild predation (consumption of eggs and immature stages of the heterospecific predator) by *A. largoensis* (**A**) and *E. alatus* (**B**) in the presence or absence of acaricide residues (abamectin) and coconut pollen. Bars represent means ± SE. Statistical significance was evaluated using aligned rank transform (ART) ANOVA. In panel A, the horizontal line and asterisk (*) indicate a significant main effect of pollen (P < 0.05). In panel B, different letters indicate significant differences among treatment combinations according to pairwise comparisons based on estimated marginal means (EMMs) with Tukey’s adjustment (P < 0.05)

The effects of pesticide residues and extraguild food (pollen) on intraguild predation differed between *A. largoensis* and *E. alatus* (Fig. 2). For *A. largoensis*, ART ANOVA detected a significant effect of pollen availability (F₁,₇₆ = 7.30, P = 0.0085), whereas neither pesticide residues (F₁,₇₆ = 0.52, P = 0.4724) nor the interaction between pesticide residues and pollen (F₁,₇₆ = 1.53, P = 0.2203) significantly affected intraguild prey consumption. In contrast, for *E. alatus*, intraguild prey consumption was significantly affected by pesticide residues (F₁,₇₆ = 44.83, P < 0.0001), pollen availability (F₁,₇₆ = 78.80, P < 0.0001), and their interaction (F₁,₇₆ = 27.16, P < 0.0001).

Overall, intraguild prey consumption by *A. largoensis* was higher in the absence of pollen than in its presence, regardless of pesticide residues (Fig. 2). For *E. alatus*, intraguild prey consumption was highest in the absence of both pesticide residues and pollen, whereas the combination of pesticide residues and pollen resulted in the lowest consumption. Intermediate levels of intraguild prey consumption were observed when either pesticide residues or pollen was present alone (Fig. 2).

**Fig. 3:**
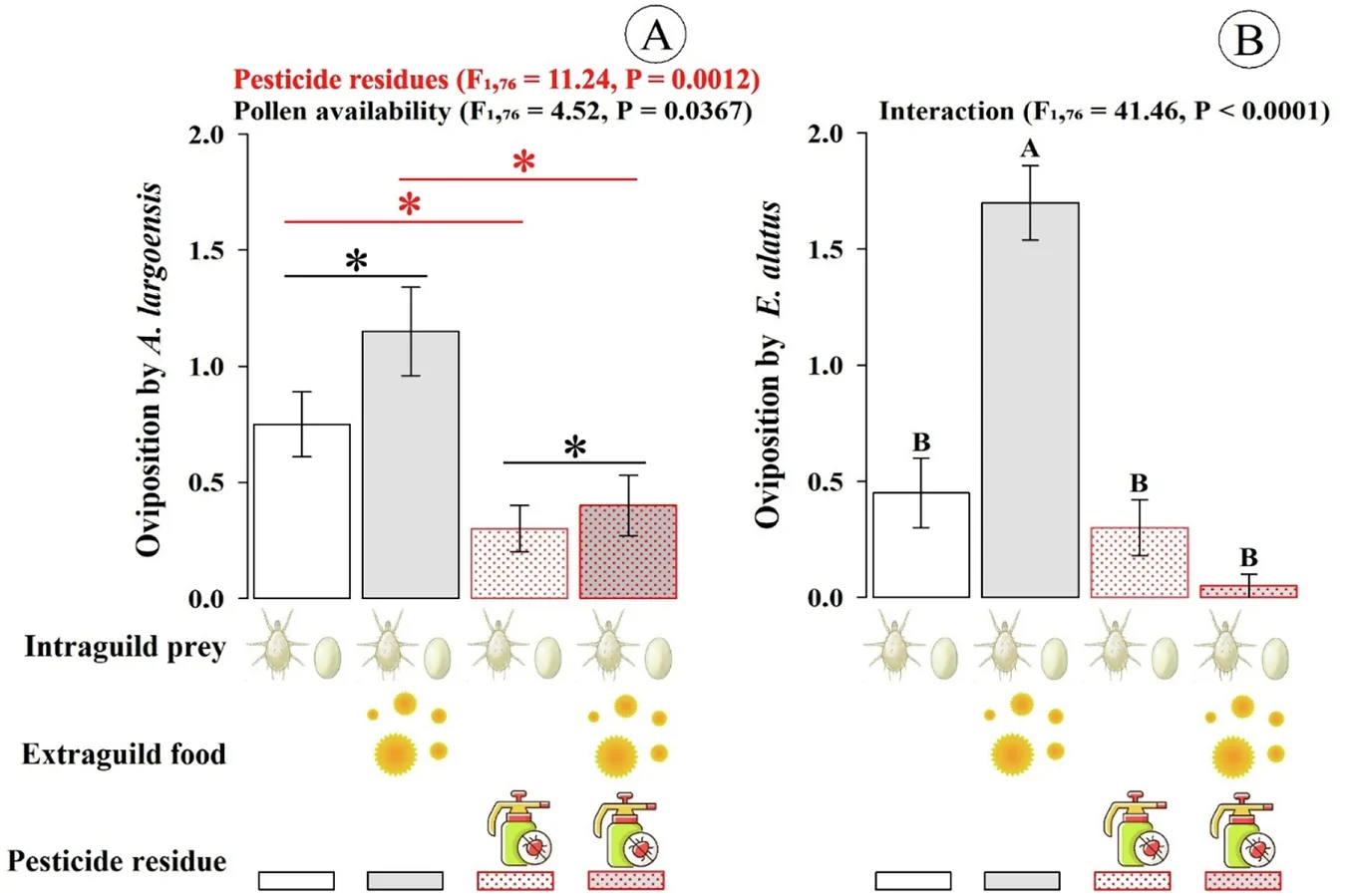
Oviposition by *A. largoensis* (**A**) and *E. alatus* (**B**) in the presence or absence of acaricide residues (abamectin) and coconut pollen. Bars represent means ± SE. Statistical significance was evaluated using aligned rank transform (ART) ANOVA. In panel A, horizontal lines and asterisks (*) indicate significant main effects of pesticide residues and pollen (P < 0.05). In panel B, different letters indicate significant differences among treatment combinations according to pairwise comparisons based on estimated marginal means (EMMs) with Tukey’s adjustment (P < 0.05)

Oviposition responses also differed between *A. largoensis* and *E. alatus* (Fig. 3). For *A. largoensis*, ART ANOVA revealed significant effects of pesticide residues (F₁,₇₆ = 11.24, P = 0.0012) and pollen availability (F₁,₇₆ = 4.52, P = 0.0367), whereas the interaction between these factors was not significant (F₁,₇₆ = 0.28, P = 0.5953). Overall, oviposition by *A. largoensis* decreased in the presence of pesticide residues and increased in the presence of pollen, with no significant interaction between these factors

For *E. alatus*, oviposition was significantly affected by pesticide residues (F₁,₇₆ = 61.36, P < 0.0001), pollen availability (F₁,₇₆ = 20.64, P < 0.0001), and their interaction (F₁,₇₆ = 41.46, P < 0.0001). Pairwise comparisons showed that oviposition was highest when pollen was available in the absence of pesticide residues, whereas the other treatment combinations showed significantly lower oviposition (Fig. 3).

### Effects of pesticide residues on the population dynamics of predators in arenas

**Fig. 4:**
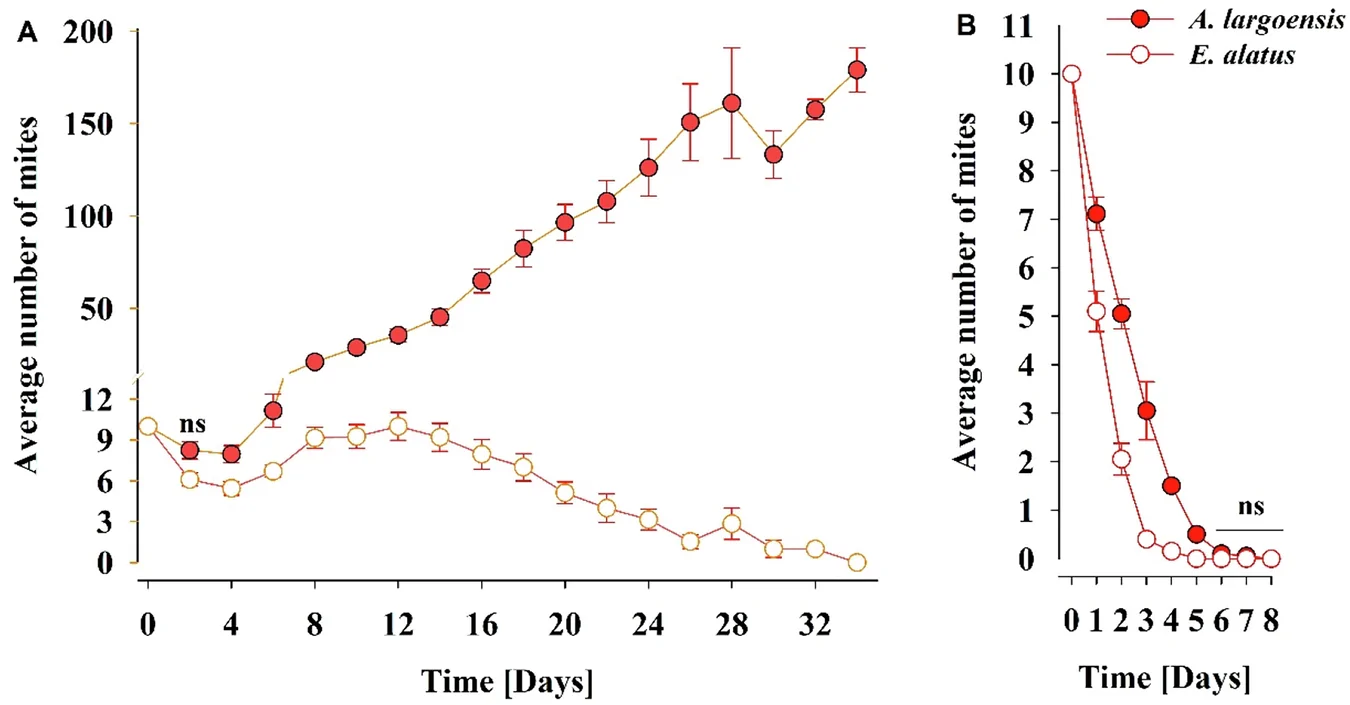
Population dynamics of *A. largoensis* and *E. alatus* in the absence **A** and presence of abamectin residues **B** and pollen. Differences between treatments were observed in all evaluations, except at time 0 and where indicated by ns (not significant, P > 0.05)

A significant effect of the interaction between treatment and time on the mean mite densities in the arenas (F1,297 = 26; P < 0.0001) was observed in the presence of pesticide residues, indicating that the population decline pattern differed between the two species. Significant effects were also observed for both the treatment factor (F1,297 = 60; P < 0.0001) and time (F1,297 = 629; P < 0.0001), reflecting a consistent reduction in population densities over the days and a greater sensitivity of *E. alatus* to the presence of pesticide residue compared to *A. largoensis*. Both predator species showed a progressive reduction in their populations over time, with *E. alatus* becoming extinct by the 5th day after the start of the experiment and *A. largoensis* by the 8th day after the start of the experiment (Fig. 4). Starting from the 6th day, the population densities of both species became equal, due to the early extinction of *E. alatus* and the rapid decline of *A. largoensis* (P ≥ 0.85).

The results of population dynamics in the absence of pesticide residues demonstrated a significant effect of the interaction between treatment and time on the average densities of mites (F1, 475 = 703; P < 0.0001), reflecting distinct population dynamics between the two predator species. Additionally, significant effects were observed for both the treatment factor (F1, 475 = 1362; P < 0.0001) and time (F1, 475 = 129; P < 0.0001), indicating clear differences in population trajectories between the species over time (Fig. 4). Both species showed a trend of decline in the first two assessments, with no difference observed only in the first (P = 0.08). However, starting from the second evaluation (day 4), *A. largoensis* began to show consistently higher population densities than *E. alatus* (P ≤ 0.02), maintaining this superiority until the end of the experiment. The populations of *E. alatus* were extinct by the 34th day.

## Discussion

The present study demonstrated that intraguild predation (IGP) occurred reciprocally between *A. largoensis* and *E. alatus*, and that both extraguild food (pollen) and pesticide residues modulated the intensity of these interactions.

Differences in feeding strategy between *A. largoensis* and *E. alatus* provide a plausible explanation for the contrasting effects of pollen on intraguild predation (Schausberger and Croft, 2000; McMurtry et al. 2013). Based on their feeding habits, microhabitat use, and morphology, *A. largoensis* is classified as a Type III-b generalist predator, whereas *E. alatus* is a Type IV predator that feeds preferentially on pollen (McMurtry et al. 2013). Consistent with these trophic strategies, the availability of pollen reduced intraguild predation in both species, but the reduction was more pronounced in *E. alatus*. In contrast, *A. largoensis* maintained relatively high levels of intraguild predation even in the presence of pollen, reflecting its greater dependence on animal prey. Pollen is a nutritionally rich alternative food source that enhances the development, survival, and reproductive performance of many phytoseiid mites (Riahi et al. 2016; Massaro et al. 2016; Tsolakis et al. 2016; Liu and Zhang, 2017; Moretti et al. 2023). Consequently, pollen supplementation has been used in biological control programs to promote the establishment and conservation of predatory mites, thereby modifying predator–prey interactions and influencing population dynamics in agricultural systems (Shakya et al. 2009; Delisle et al. 2015; Gurr et al. 2017; Calabuig et al. 2018; Lee and Zhang, 2018).

Intrinsic factors, such as body size, may also influence predator–predator interactions (Claessen et al. 2002). *Amblyseius largoensis* is generally larger than *E. alatus* (Lofego et al. 2024), which likely confers an advantage in aggressive encounters and may explain its dominance in population dynamics experiments. Smaller predators often face higher handling costs or are unable to attack heterospecific adults effectively (Famah-Sourassou et al. 2013), which could have contributed to the exclusion of *E. alatus* despite its ability to prey upon eggs and larvae of *A. largoensis*.

Beyond the classic view of intraguild predation leading to competitive exclusion, our results demonstrate that chemical stressors can act as additional modulators of these ecological processes. Abamectin exposure not only altered direct predation and reproduction but also reshaped the trajectory of species coexistence, showing that contaminants can influence community outcomes that would otherwise be determined by intrinsic biological traits alone. Such changes exemplify how anthropogenic chemicals can modify the direction and strength of ecological interactions within multitrophic systems.

Sublethal exposure to abamectin altered both reproductive performance and intraguild predation, reducing oviposition in both predator species and differentially affecting predation, with *E. alatus* being more sensitive than *A. largoensis*. These results are consistent with previous studies demonstrating that sublethal pesticide exposure impairs foraging, handling time, and reproduction in phytoseiid mites (Lima et al. 2013a, 2015; Barros et al. 2022). The greater sensitivity of *E. alatus* may further increase its competitive disadvantage relative to *A. largoensis* in agroecosystems where abamectin is routinely applied. More importantly, our findings demonstrate that sublethal pesticide exposure can modify ecological interactions between predators, rather than simply affecting individual performance. By altering both behavioral (intraguild predation) and demographic (oviposition) processes, abamectin has the potential to influence species coexistence and trophic interactions, reinforcing the ecotoxicological perspective that pesticides act not only as toxic agents but also as ecological stressors capable of restructuring food webs.

Intraguild predation is a natural phenomenon in ecosystems and can strongly influence the effectiveness of pest biological control. The present study suggests a possible displacement of *E. alatus* by *A. largoensis* in the absence of pesticide residues and in the presence of extraguild food (pollen). Both predators are reported as natural enemies of *A. guerreronis* during the dispersal process of this pest (Galvão et al. 2007; Melo et al. 2009) and co-occur on coconut leaflets (Gondim and de Moraes, 2001). However, studies on coconut acarofauna consistently indicate higher densities of *A. largoensis* than *E. alatus* (Lawson-Balagbo et al. 2008), which may be explained not only by intraguild predation but also by species-specific traits such as feeding strategy and body size. While *E. alatus* is a type IV predator that relies heavily on pollen, *A. largoensis* is a type III predator more dependent on animal prey (McMurtry et al. 2013), and its larger body size (Galvão et al. 2007) likely provides an advantage in direct interactions. These ecological traits reinforce the asymmetry observed in our experiments. Additionally, one of the main pesticides used against *A. guerreronis* is abamectin, and in systems where this product is applied, the persistence of both predators may be compromised, potentially leading to the extinction of one or both species.

On the other hand, the architecture of the coconut palm canopy, characterized by leaves and fruit bunches arranged in a spiral pattern (approximately 120° apart) and partially overlapping, may hinder the uniform deposition of pesticide sprays (Sobral, 1998). Consequently, mites inhabiting different parts of the canopy may be exposed to heterogeneous levels of pesticide residues. Such spatial variation in exposure may contribute to the persistence of predatory mite populations even in sprayed areas, although this hypothesis should be confirmed under field conditions. However, even under heterogeneous exposure to pesticide residues, species-specific ecological traits are likely to influence competitive outcomes. Considering its broader diet, larger body size, and lower sensitivity to abamectin compared with *E. alatus*, *A. largoensis* appears to have a competitive advantage, which may help explain its consistent dominance on coconut leaflets.

The results of the present study demonstrate that chemical stressors such as abamectin can cascade from behavioral impairments to population-level shifts, with implications for ecosystem services such as biological control. From an applied perspective, these findings suggest that pesticide selectivity should be evaluated not only in terms of predator survival but also in relation to its effects on ecological interactions among natural enemies. Preserving these interactions is essential for maintaining the effectiveness and long-term stability of biological control programs within integrated pest management (IPM) strategies. Nevertheless, further field studies are needed to assess the actual impact of abamectin residues under natural conditions.

From an ecotoxicological perspective, these findings highlight that sublethal pesticide exposure can alter ecological processes that are rarely considered in conventional risk assessments. By influencing predator–predator interactions and community composition, chemical stressors may induce indirect effects that extend beyond individual toxicity. Incorporating such interaction-based endpoints into ecotoxicological evaluations could improve our understanding of the real ecological risks posed by pesticides in agroecosystems. Such an approach would contribute to the development of pesticide risk assessment frameworks that better support sustainable pest management and the conservation of beneficial arthropods in agricultural systems.

## Data Availability

The experimental data of this study are available in https://doi.org/10.5281/zenodo.16551361.
